# The Concentration of Organic Acids in Cranberry Juice Modulates the Gut Microbiota in Mice

**DOI:** 10.3390/ijms222111537

**Published:** 2021-10-26

**Authors:** Valentine Renaud, Vanessa P. Houde, Geneviève Pilon, Thibault V. Varin, Cyril Roblet, André Marette, Yvan Boutin, Laurent Bazinet

**Affiliations:** 1 Institut sur la Nutrition et les Aliments Fonctionnels (INAF), Université Laval, Québec, QC G1V 0A6, Canada; valentine.renaud.1@ulaval.ca (V.R.); vanessa.houde.1@ulaval.ca (V.P.H.); genevieve.pilon@criucpq.ulaval.ca (G.P.); thibaut.varin.1@ulaval.ca (T.V.V.); andre.marette@criucpq.ulaval.ca (A.M.); yvan.boutin@tbt.qc.ca (Y.B.); 2 Laboratoire de Transformation Alimentaire et Procédés ElectroMembranaires (LTAPEM, Laboratory of Food Processing and ElectroMembrane Processes), Université Laval, Québec, QC G1V 0A6, Canada; 3 Québec Heart and Lung Institute, Department of Medicine, Université Laval, Québec, QC G1V 4G5, Canada; 4 Fruit d’Or, Villeroy, QC G0S 3K0, Canada; croblet@fruit-dor.ca; 5 TransBioTech, Lévis, QC G6V 6Z3, Canada

**Keywords:** cranberry juice, polyphenols, organic acids, organic acid removal, electrodialysis with bipolar membrane, mice model, intestinal inflammation, gut microbiota

## Abstract

A daily consumption of cranberry juice (CJ) is linked to many beneficial health effects due to its richness in polyphenols but could also awake some intestinal discomforts due to its organic acid content and possibly lead to intestinal inflammation. Additionally, the impact of such a juice on the gut microbiota is still unknown. Thus, this study aimed to determine the impacts of a daily consumption of CJ and its successive deacidification on the intestinal inflammation and on the gut microbiota in mice. Four deacidified CJs (DCJs) (deacidification rates of 0, 40, 60, and 80%) were produced by electrodialysis with bipolar membrane (EDBM) and administered to C57BL/6J mice for four weeks, while the diet (CHOW) and the water were *ad libitum*. Different parameters were measured to determine intestinal inflammation when the gut microbiota was profiled. Treatment with a 0% DCJ did not induce intestinal inflammation but increased the gut microbiota diversity and induced a modulation of its functions in comparison with control (water). The effect of the removal of the organic acid content of CJ on the decrease of intestinal inflammation could not be observed. However, deacidification by EDBM of CJ induced an additional increase, in comparison with a 0% DCJ, in the *Lachnospiraceae* family which have beneficial effects and functions associated with protection of the intestine: the lower the organic acid content, the more bacteria of the *Lachnospiraceae* family and functions having a positive impact on the gut microbiota.

## 1. Introduction

Over the past years, there has been a growing interest in cranberry juice (CJ) due to its composition. Indeed, this type of juice is rich in polyphenols and more particularly in anthocyanins and proanthocyanidins (PACs). Six major anthocyanins are present in CJ and are found in the form of aglycons of cyanidin and peonidin conjugated with arabinose, galactose, and glucose [1,2,3,4]. In regard to PACs, monomers, 2–3mers, 4–6mers, 7–10mers, and polymers have been detected in CJ [1,5,6,7]. On the other hand, three major organic acids are found at high concentrations in CJ, namely, citric, quinic, and malic acids and give to the juice its great titratable acidity [1,8].

A daily consumption of CJ is known to induce several effects on health. Indeed, the polyphenol content of CJ has well demonstrated its protective effect against numerous bacteria (urinary tract infections, gastric ulcers, dental plaque, etc.), its capacity in reducing the risk of developing cardiovascular disease, its anticancer effects, or the protection of cells against oxidative stress [9,10,11,12,13,14,15,16,17]. Unfortunately, the high content in organic acids of this juice could also cause undesirable intestinal side effects in some cases. Indeed, between 2 and 29% of withdrawal were reported in some clinical studies carried out on the beneficial health effects of CJ daily consumed [18,19,20,21,22,23]. It has also been demonstrated in vitro, that the content in citric acid of CJ was responsible for the disruption of intestinal Caco-2 cell barrier integrity [7,24]. Our previous pilot study indicated that a daily consumption of a concentrated (14°B) and a 1:1 (7°B) CJ induced symptoms of intestinal inflammation in a C57BL/6J mouse model [25]. Furthermore, the CJ composition could induce a modulation of the gut microbiota. Indeed, the positive effect of polyphenols of many plant sources on the gut microbiota has already been investigated [26,27,28,29,30,31]. On the other hand, organic acids are known as bacterial growth inhibitors and are used as acidifiers in the diet of livestock, but their effect is mitigated [32,33,34,35].

In order to prevent such inflammatory side effects associated with the consumption of CJ while preserving the beneficial effects of polyphenols, an electromembrane process has been developed [36,37]. This process is named electrodialysis with bipolar membrane (EDBM) and decreases selectively and drastically the content in organic acids while preserving the polyphenol content in CJ. The principle of EDBM is based on the use of bipolar membranes leading to the dissociation of water molecules into H^+^ and OH^−^ ions, under the effect of a current application [38,39]. This green and ecofriendly process has demonstrated the effect of global organic acid removal on the protection against disruption of in vitro intestinal Caco-2 cell barrier integrity. Additionally, it has been highlighted that citric acid present in CJ is the main organic acid responsible for the disruption of the intestinal epithelial barrier in vitro [7,24]. However, to the best of our knowledge, the effects of removing organic acids by deacidification of CJ by EDBM, at different levels, its protective effect against intestinal inflammation during daily consumption in mice, and its capacity to modulate the composition and the functions of the gut microbiota, had never been studied.

In this context, the aim of the present work was to study the impacts of a CJ at different concentrations in organic acid on intestinal inflammation and gut microbiota.

## 2. Results

### 2.1. Cranberry Juices Composition

The electrodialytic process induced changes in term of pH, conductivity and titrata-ble acidity of the CJ as the rate of deacidification increased (Table 1). Indeed, the initial pH of the CJ was 2.59 ± 0.03 and the pH increased by 4, 10, and 20%, respectively, for the 40, 60, and 80% DCJ (*p* < 0.0001). The initial conductivity of the CJ was 2 409.00 ± 5.00 µS/cm and statistically decreased during the electrodialytic process, by 22, 18, and 24%, respectively, for the 40, 60, and 80% deacidified CJ (DCJ) (*p* < 0.0001). The initial titratable acidity of the CJ was 9.25 ± 0.05 g (of citric acid eq/L) and it decreased by 42, 60, and 79%, respectively, for the 40, 60, and 80% DJC (*p* < 0.0001).

Three major organic acids were detected in the 0% DCJ, the most abundant was citric acid (11.59 ± 0.20 g/L), then quinic acid (10.35 ± 0.31 g/L) and malic acid (6.03 ± 0.10 g/L). As expected, the EDBM induced statistically significant decreases of the content of citric and malic acid according to the rate of deacidification. Indeed, the content of citric acid was statistically reduced by 41, 60, and 80%, respectively, for the 40, 60, and 80% DCJs and the content of malic acid was statistically reduced by 60, 78, and 100%, respectively, for the 40, 60, and 80% DCJs (*p* < 0.0001 for both organic acids). The content of quinic acid remained stable and did not statistically change between the 0, 40, and 60% DCJs but it statistically decreased by 11% in the 80% DJC compared to the 0% DJC (*p* = 0.0002).

Six anthocyanins were detected in the 0% DCJ and were also detected in the 40, 60, and 80% DCJs. The anthocyanin content seemed to be slightly affected by the electrodialytic process according to the type of anthocyanin and the rate of deacidification of CJ, compared to the 0% DCJ. There were no statistically significant changes in the content of C-3-galactoside, C-3-arabinoside, P-3-galactoside, P-3-arabinoside, and the total content of anthocyanins between the 0, 40, and 60% DCJ while they statistically decreased in the 80% DCJ (respectively, *p* = 0.001, *p* = 0.0012, *p* = 0.0006, *p* = 0.0002, and *p* = 0.001). Additionally, there were some statistically significant changes in the content of C-3-glucoside and P-3-glucoside according to the rate of deacidification. There was a significant increase of C-3-glucoside content of the 40% DCJ compared to the 60% DCJ and a significant increase of P-3-glucoside content of the 40% DCJ compared to the 0 and 80% DCJs (*p* = 0.0207 and *p* = 0.0052).

PACs were also detected in the 0, 40, 60, and 80% DCJs. The electrodialytic process did not induce statistically significant changes in the content of monomers, 2–3 mers, 4–6 mers, 7–10 mers, polymers, and the total content of PACs between the 0, 40, 60, and 80% DCJs (respectively, *p* = 0.3137, *p* = 0.2426, *p* = 0.4669, *p* = 0.4649, *p* = 0.4649, and *p* = 0.3752).

The total polyphenol content was slightly affected by the electrodialytic process. In-deed, the total polyphenol content of the 40 and 80% DCJs was statistically decreased compared to the 0 and 60% DCJs (*p* = 0.0239).

### 2.2. Animals

#### 2.2.1. Effect of DCJ on the Food Intake and Body Weight

**Figure 1:**
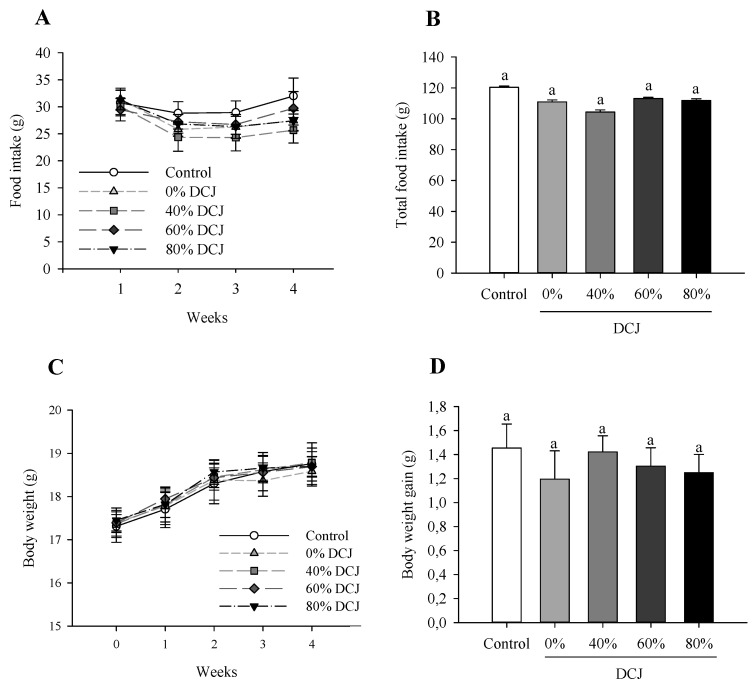
Effect of the deacidification of CJ on the (**A**) food intake, (**B**) total food intake, (**C**) body weight, and (**D**) total body weight gain in mice in comparison with a control (water). Results are presented as the mean ± SEM and *n* = 8 for **A** and **B** and *n* = 12 for **C** and **D**. ANOVAs combined with a Tukey’s test were done on **A**–**D**. For (**A**,**C**), the absence of letter means that there is no statistically significant effect at a probability level of 0.05. For (**B**,**D**), a different letter means that the results are significantly different at a probability level of 0.05.

As depicted in Figure 1A, there was no significant effect of the level of deacidification of CJ on the food intake on T1, T2, T3, and T4. Additionally, considering the total food intake, there were no statistically significant differences between the control, 0, 40, 60, and 80% DCJ groups (Figure 1B).

When comparing the body weight of the different DCJ groups to the control group and at one time of measurement, statistical analyses revealed that there were no statistically significant effects of the deacidification percentage of the DCJ (Figure 1C). Considering the total body weight gain, statistical analyses revealed that there were no statistically significant differences between the five groups of mice (Figure 1D).

#### 2.2.2. Feces Analyses

At T0, the Hemoccult Sensa test did not reveal any presence of occult blood (OB) in the feces of the control, 0, 40, 60, and 80% DCJ groups. This was consistent since all groups of mice received only water. At T2 and T4, there was no presence of OB in the feces of the control group. This was consistent since the mice of the control group received only water during the experiment but interestingly, the 0, 40, 60, and 80% DCJ groups did not show presence of OB in their feces.

#### 2.2.3. *Post-Mortem* Observations

The daily consumption of the different DCJs did not impact the weight of the liver, the spleen and the colon, and the length of the colon, when compared to the control (Data not shown).

Inspired from existing literature, macroscopic observations were carried out on the ileum and the colon of the different groups of mice [40,41] (Table 2A,B). In the ileum, only inflammation and vascularization were observed and were statistically different between the groups (*p* = 0.0007 and *p* = 0.0148). Indeed, the scores of inflammation were higher for the 0, 40, and 60% DCJ groups when the scores of vascularization indicated that the 0% DCJ group had the higher score and it was statistically different from the other groups. Regarding the total score, the 40 and 60% DCJ groups were statistically different from the control and 80% DCJ groups (*p* = 0.0017).

In the colon, inflammation, vascularization, and thickening were observed. Statistical analyses of the scores of inflammation and thickening revealed that there was no statistically significant difference between the control, 0, 40, 60, and 80% DCJ groups. For the vascularization, the score of the 60% DCJ group was statistically different, but really close to the probability level of 5%, from the control, 0, 40, and 80% DCJ groups (*p* = 0.0484). Regarding the total score, there was no statistically significant difference between the different groups.

#### 2.2.4. Gene Expression by qRT-PCR

**Figure 2:**
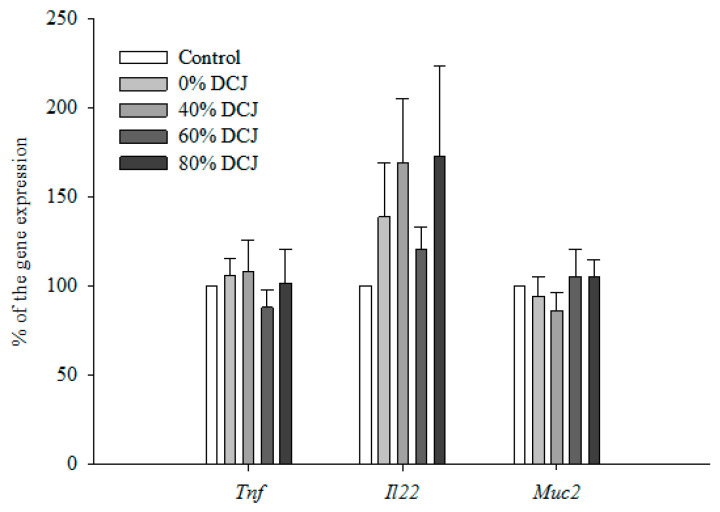
Effect of the deacidification of CJ on the expression of *Tnf*, *Il22*, and *Muc2*. Results are presented in % of the gene expression ± SEM compared to the control (*n* = 12). A Student’s t test was performed to evaluate the differences between the treated groups and the control group.

Expression of Tumor Necrosis Factor-α (*Tnf*) in the colon was not statistically different between the 0, 40, 60, and 80% DCJ groups compared to the control group (Figure 2). Additionally, data concerning the expression of Interleukin-22 (*Il22*) in the colon were not statistically different between the 0, 40, 60, and 80% DCJ groups compared to the control group (Figure 2). Concerning the expression of Mucin2 (*Muc2*) in the colon, no statistical differences were observed between the 0, 40, 60, and 80% DCJ groups compared to the control group (Figure 2).

#### 2.2.5. Impacts of DCJ on the Gut Microbiota

##### Effect of DCJ Administration on the Composition and the Functions of the Gut Microbiota

**Figure 3:**
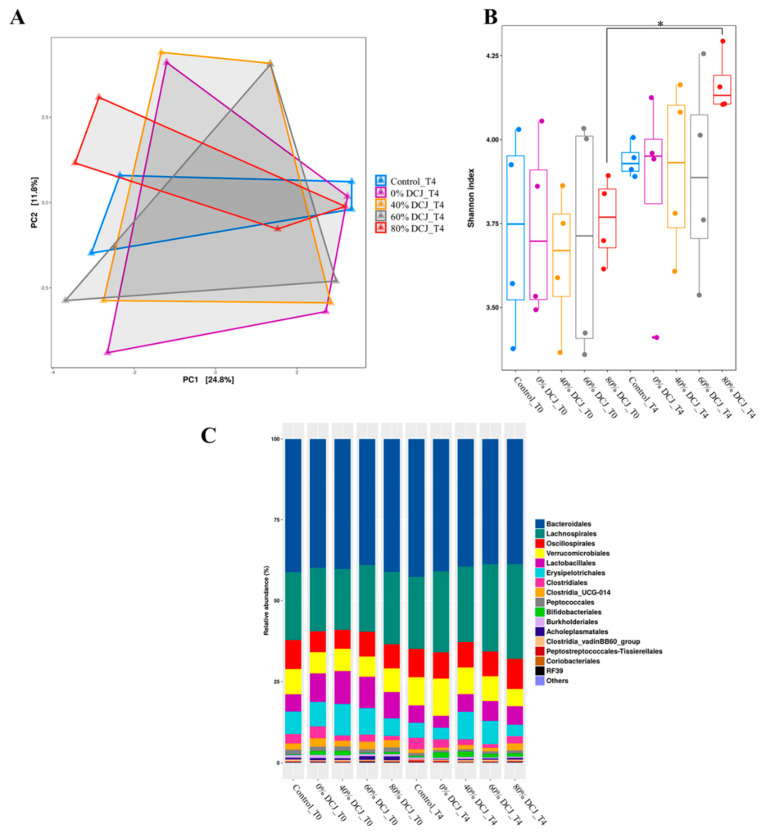
Impact of the administration of the different DCJs on (**A**) the global structure of the microbiota after 4 weeks of treatment, reflected by the principal coordinate analysis (PcoA) on the unweighted UniFrac (Unique Fraction) distance matrix. (**B**) The alpha-diversity of the gut microbiota before (T0) and after the end (T4) of the experiment, as measured with the Shannon index. (**C**) Relative abundance of taxa at the genus level.

Fecal samples were collected before (T0) and at the end (T4) of the experiment to investigate the impact of the different DCJs on the gut microbiota. Principal coordinate analysis (PcoA) revealed no clear separation between the microbial communities of the different groups (Figure 3A). Alpha diversity was further estimated by measuring the Shannon index for each group (Figure 3B). After 4 weeks of treatment, an increase of the diversity index was only observed in the 80% DCJ group while the Shannon index for the control, 0, 40, and 60% DCJ groups remained unchanged during experimentation. Measurements of the relative abundance of taxa at the genus revealed an increased abundance of *Lachnospirales* in the gut microbiota of the DCJ groups after the 4-weeks experiment (Figure 3C).

**Figure 4:**
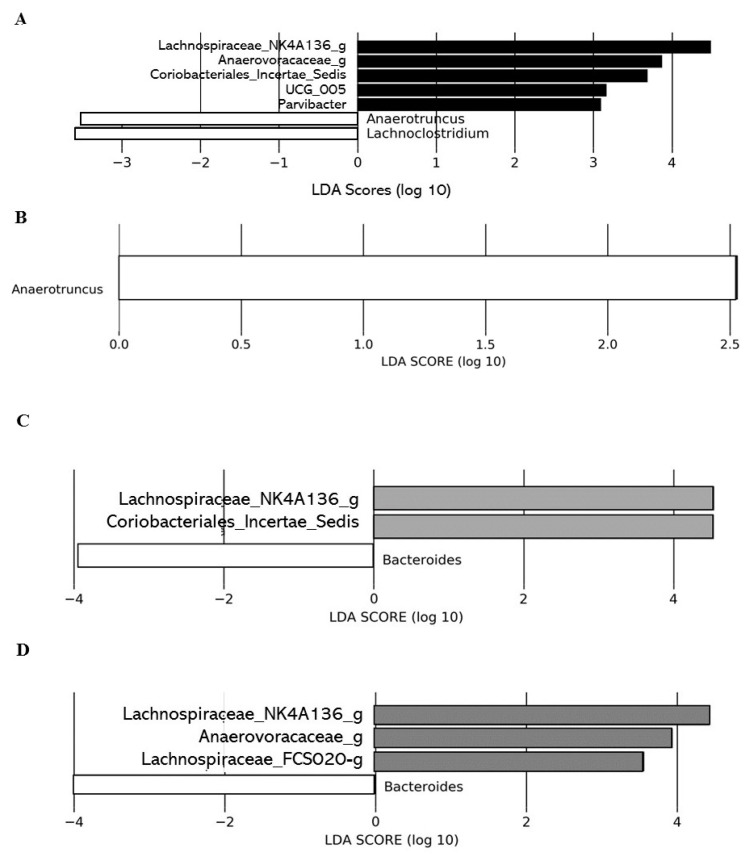
Administration of the different DCJs is associated with changes in the gut microbial of mice in comparison with the control (water). The linear discriminant analysis (LDA) effect size was calculated in order to explore the taxa within genus levels that more strongly discriminated between the gut microbiota of mice treated with (**A**) water (white) and 0% DCJ (dark), (**B**) water and 40% DCJ (light grey), (**C**) water and 60% DCJ (medium grey), and (**D**) water and 80% DCJ (dark grey). Families followed by the label ‘-g’ indicate unidentified genera.

Linear discriminant analysis effect size (LEfSe) analysis conducted on T0 did not show significant differences in the gut microbial communities between the control group and the different DCJ-treated mice (data not shown). However, LEfSe analysis on T4 highlighted significant differences in the gut microbial communities of 0% DCJ-treated mice from those of control mice characterized by an increase of *Lachnospiraceae_NK4A136_group*, *UCG_005*, *Parvibacter*, *Anaerovoracaceae_g*, *Coriobacteriales_Incertae_Sedis_g*, and by a lower representation of taxa assigned to the genera *Anaerotruncus* and *Lachnoclostridium* (Figure 4A). Overrepresentation of the genera *Anaerotruncus* was identified as the main feature discriminating control mice from 40% DCJ-treated mice microbiota (Figure 4B). Furthermore, there was an increased presence of families *Lachnospiraceae_NK4A136_g* and *Coriobacteriales_Incertae_Sedis_g* and a lower presence of *Bacteroides thetaiotamicron* for the 60% DCJ-treated mice compared to the control mice (Figure 4C). Finally, the gut microbial community of the 80% DCJ-treated mice was characterized by an increase presence *of Lachnospiraceae_NK4A136_g*, *Anaerovoracaceae_g* and *Lachnospiraceae_FCS020_g* and by a lower representation of the genus *Bacteroides* (Figure 4D), whose only species identified in this study was *Bacteroides thetaiotamicron*.

**Figure 5:**
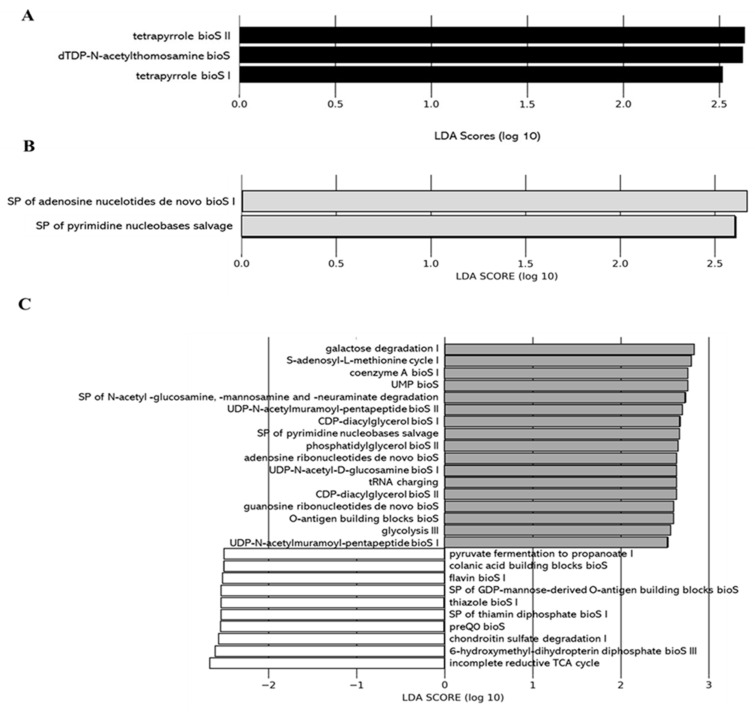

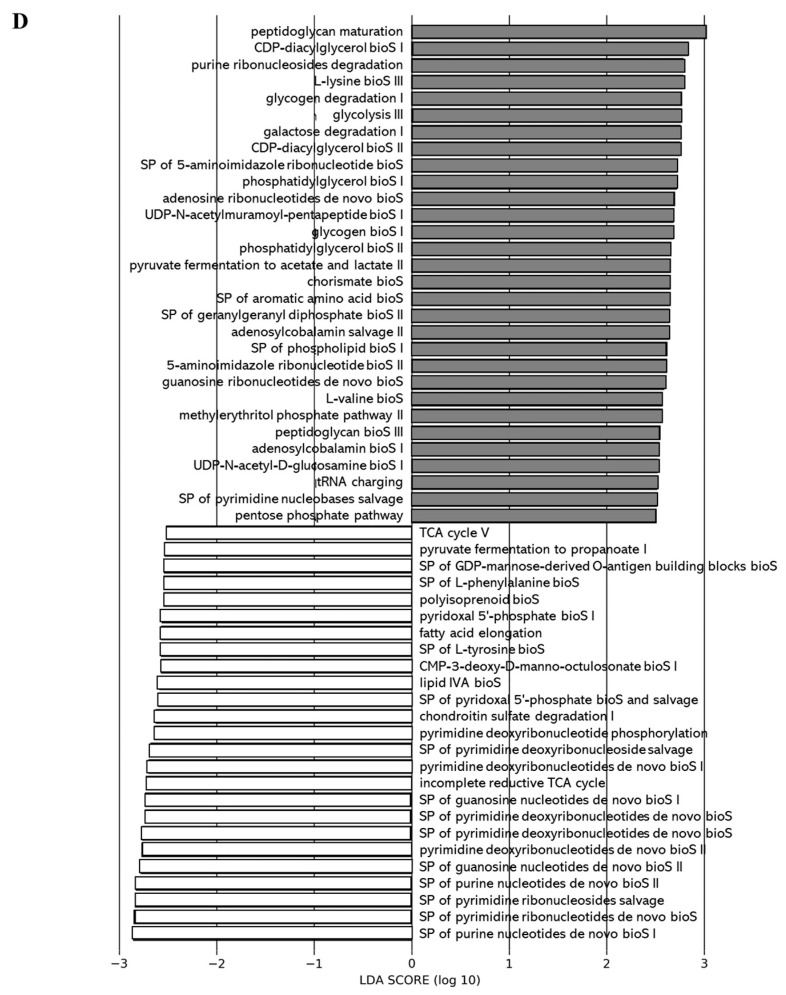
Administration of the different DCJs alter metabolic pathways in the gut microbiota of mice in comparison with the control (water). The linear discriminant analysis (LDA) effect size was calculated in order to explore the microbial functions that more strongly discriminated between the gut microbiota of mice treated with (**A**) water (white) and 0% DCJ (dark), (**B**) water and 40% DCJ (light grey), (**C**) water and 60% DCJ (medium grey), and (**D**) water and 80% DCJ (dark grey). bioS: biosynthesis, SP: superpathway.

We applied the Phylogenetic Investigation of Communities by Reconstruction of Unobserved States 2 (PICRUSt2) pipeline to assess if functional alterations in the gut microbiome induced by the different juices as compared to the control (water) could be observed. Functions associated with tetrapyrrole biosynthesis (e.g., tetrapyrrole bioS I and II) and sugar nucleotide biosynthesis (e.g., dTDP-N-acetylthomosamine bioS) were more represented in the gut microbiota of the 0% DCJ-treated mice than in control mice (Figure 5A). Functions of nucleotide biosynthesis (e.g., SP of adenosine nucleotides *de novo* bioS I) were more represented in the gut microbiota of the 40% DCJ-treated mice than the control mice (Figure 5B). The gut microbiota of the 60% DCJ-treated mice represented mainly functions of degradation (e.g., galactose, amine, and polyamine degradation) and nucleotide, pentapeptide, phospholipid, sugar, and carrier biosynthesis (e.g., UMP bioS, UDP-N-acetylmuramoyl-pentapeptide bioS I, CDP-diacylglycerol bioS I, UDP-N-acetyl-D-glucosamine bioS I, and coenzyme A bioS I) compared to the control mice (Figure 5C). Additionally, functions of degradation (e.g., purine ribonucleoside, glycogen, galactose, and carboxylate degradation), phospholipid, amino acid, nucleotide, pentapeptide, polyprenyl, and enzyme cofactor biosynthesis (e.g., CDP-diacylglycerol bioS I, L-lysine bioS III, adenosine ribonucleotides *de novo* bioS, UDP-N-acetylmuramoyl-pentapeptide bioS I, SP of geranylgeranyl diphosphate (GGPP) bioS II, and andenosylcobalamin bioS), generation of precursor metabolites and energy (e.g., pentose phosphate pathway) were more represented in the gut microbiota of the 80% DCJ-treated mice (Figure 5D). It could be noted that at T0, some differences were observed between the control group and the 0, 60, and 80% DCJs but the functions observed at this time were not found at T4 (data not shown).

##### Effect of Organic Acid Removal on the Composition and the Functions of the Gut Microbiota

**Figure 6:**
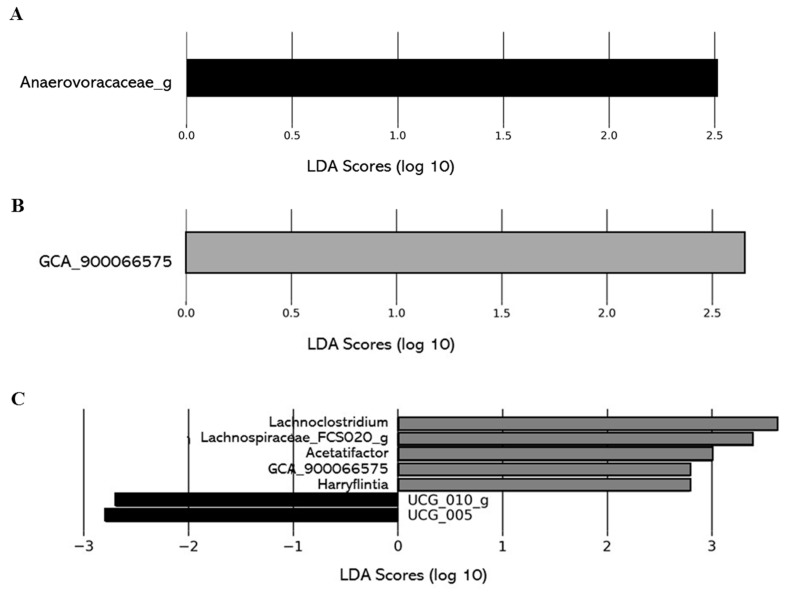
Level of deacidification of CJ is associated with changes in the gut microbial of mice. The linear discriminant analysis (LDA) effect size was calculated in order to explore the taxa within genus levels that more strongly discriminated between the gut microbiota of mice treated with (**A**) 0% DCJ (dark) and 40% DCJ (light grey), (**B**) 0% DCJ and 60% DCJ (medium grey), and (**C**) 0% DCJ and 80% DCJ (dark grey). Families followed by the label ‘-g’ indicate unidentified genera.

The impact of the level of deacidification of CJ on the composition of the gut microbiota was also investigated comparing the gut microbiota composition of the 0% DCJ-treated mice to the 40, 60, and 80% DCJ groups. On T4, overrepresentation of the family *Anaerovoracaceae_g* was identified as the main feature discriminating 0% DCJ-treated mice from 40% DCJ-treated mice metagenomes, whereas an increased presence of *GCA_900066575* was characteristic gut microbial feature of 60% DCJ-treated mice vs. 0% DCJ-treated mice (Figure 6A,B). Finally, there was an increased presence of *Lachnoclostridium*, *Acetatifactor muris*, *GCA_900066575*, *Harryflintia*, *Lachnospiraceae_FCS020_g*, and a lower presence of *UCG_010_g* and *UCG_005* for the 80% DCJ-treated mice vs. the 0% DCJ-treated group (Figure 6C). On T0, some differences in the gut community could be observed but they were not found on T4 (data not shown).

**Figure 7:**
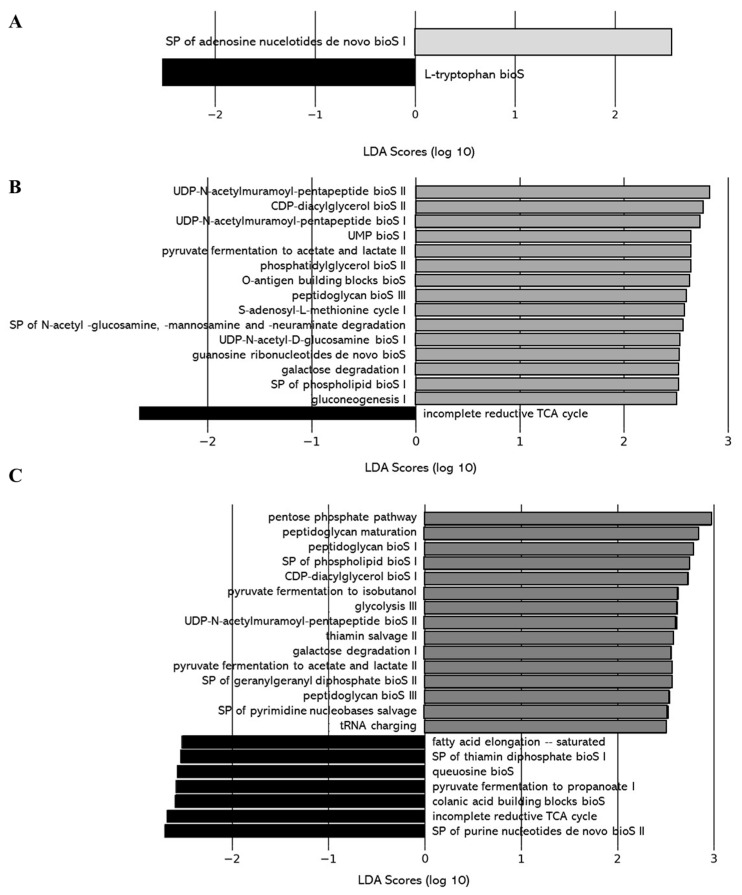
Level of deacidification of CJ is associated with altered metabolic pathways in the gut microbiota of mice. The linear discriminant analysis (LDA) effect size was calculated in order to explore the microbial functions that more strongly discriminated between the gut microbiota of mice treated with (**A**) 0% DCJ (dark) and 40% DCJ (light grey), (**B**) 0% DCJ and 60% DCJ (medium grey), and (**C**) 0% DCJ and 80% DCJ (dark grey). bioS: biosynthesis, SP: superpathway.

As for the gut microbiota composition, the impact of the level of deacidification of CJ on the functions of the gut microbiota was compared between the 0% DCJ-treated mice and the 40, 60, and 80% DCJ groups. Function of purine nucleotide *de novo* biosynthesis (e.g., SP of adenosine nucleotides *de novo* bioS I) was more represented in the gut microbiota of the 40% DCJ-treated mice while function of L-tryptophan biosynthesis was more represented in the gut microbiota of the 0% DCJ-treated mice (Figure 7A). The gut microbiota of the 60% DCJ-treated mice represented mainly functions of degradation (e.g., carboxylate, galactose, amines, and polyamines degradation) and carbohydrate, lipid, and peptidoglycan biosynthesis (e.g., gluconeogenesis I, SP of phospholipid bioS I, and UDP-N-acetylmuramoyl-pentapeptide bioS I) compared to the 0% DCJ-treated mice (Figure 7B). Additionally, functions of degradation (e.g., galactose and amine and polyamine degradation), carbohydrate, lipid, peptidoglycan, and polyprenyl biosynthesis (e.g., glycolysis III, SP of phospholipid bioS I, peptidoglycan bioS I, and SP of GGPP bioS II), generation of precursor metabolites and energy (e.g., pentose phosphate pathway I) were more represented in the gut microbiota of the 80% DCJ-treated mice than in the 0% DCJ-treated mice (Figure 7C). As for the gut microbiota composition, there were small differences in the functions exhibited in the gut of mice receiving the 40, 60, and 80% DCJs compared to the 0% DCJ group on T0 but these functions were not found on T4 (data not shown).

## 3. Discussion

Considering the results of the physicochemical composition of the DCJs, these were in accordance with the literature. Indeed, as the rate of deacidification increased, the EDBM induced a slight increase of the pH and decreased the conductivity and titratable acidity of the DCJs [37,42,43]. Additionally, and as expected, the EDBM induced a decrease in the organic acid content and this was consistent with the data found in the literature, the organic acids migrated according to their chemical structures and pKas, following the duration of the electrodialytic process [7,37,42]. Concerning the polyphenol content, the anthocyanins seemed to be slightly affected by the electrodialytic process according to their type and the rate of deacidification (80%) of CJ. Indeed, for the C-3-glucoside and P-3-glucoside, their initial content was very low and the sensitivity of the method of detection might have highlighted some differences, but regarding the content values, it could be assumed that they were not real. Additionally, at the end of the process, there was some black fouling on the membranes of the electrodialytic cell that was probably caused by the anthocyanins present in CJ and might explain the very slight decrease of their content observed in the 80% DCJ. The PAC content did not change during the EDBM and this was in accordance with the literature since PACs do not migrate through anionic exchange membrane [37]. Finally, the total polyphenol content was slightly affected by the process but regarding the values, these differences were very low, and it could be assumed that they were not real.

The effect of a daily consumption of CJ on the apparition of intestinal inflammation and its decrease in symptoms by further removal of organic acid content was investigated. The results indicated that there was poor evidence of intestinal inflammation induced by the 0% DCJ. Indeed, no changes in the food intake and the body weight of mice were observed. This was interesting since polyphenols, citrate, and hydroxycitrate could increase the intensity and duration of satiety, thus, modulating these two physiological parameters [44,45,46]. The 0% DCJ did not induce presence of OB in the feces of mice, and this was surprising and in contradiction with our previous results [25]. It could be hypothesized that the polyphenol content acted to protect the intestine as already observed in diet-induced obesity mice model, where inflammation was caused by the diet [28,47]. Additionally, the post-mortem analyses revealed that the 0% DCJ did not induce changes in the weight and length of the organs as already observed in our previous work [25]. The further macroscopic observations of the ileum and the colon revealed that there were no trends in the results. The total score of the 0% DCJ group was not different from that of the control group. It could be noted that the scores of the 80% DCJ were always the lowest and the specificity of this DCJ was that, after the deacidification process, it did not contain malic acid anymore and this might have an impact on the inflammation, vascularization, and thickening of the ileum and colon. Unfortunately, there was poor literature reporting the mechanisms underlying the effect of this organic acid on these organs. Finally, the analysis of the gene expression of *Tnf*, *Il22*, and *Muc2*, which are implicated in inflammatory/protection mechanisms in the colon was assessed. TNF is a pro-inflammatory cytokine predominantly produced by macrophages as well as tumor cells and initiates chronic inflammation [48]. This cytokine regulates cell survival, proliferation, cell death, plays a critical role for the maintenance of intestinal homeostasis, and promotes inflammatory responses that can be associated with many autoimmune disorders [49]. Il22 is also a cytokine, its expression in the colon is elicited under inflammatory conditions and this gene is implicated in both protective effect and pro-inflammatory response [50,51,52]. Muc2 is responsible for the production of intestinal mucus and plays a critical protective role on the epithelial barrier [53]. The results revealed that the DCJs did not modulate their expression. Limitations within the study could be acknowledged. Indeed, there was no existing literature on intestinal inflammatory effect of organic acids present in CJ in terms of their nature and concentration in the juice. Furthermore, the clinical studies presenting withdrawal of patients consuming CJ daily, did not mention the diet of participants during the study. The apparition of intestinal discomforts could then have been due to other factors than the CJ itself.

As mentioned before, these results were interesting and surprising since our previous study aiming to determine the organic acid concentration of CJ inducing intestinal inflammatory symptoms in mice showed different results [25]. Indeed, and in contrast to the 0% DCJ, a daily consumption of a 1:1 CJ (a 7°B CJ from a different lot) induced some intestinal inflammatory symptoms in mice. In order to understand such differences, a comparison of the physicochemical composition of both CJs (1:1 and 0% DCJ) was assessed and statistical analyses revealed some statistically significant differences between both CJs (see Supplementary Materials files Table S1). The organic acid content of the 0% DCJ was lower but only the malic acid content was statistically different from the 1:1 CJ. However, the major differences between both CJs were observed in the polyphenol content. Indeed, there were significant higher contents of most of anthocyanins and PACs of the 0% DCJ compared to the 1:1 CJ as well as the total polyphenol content. Such differences in composition could be explained by the fact that the two CJs were from different lots and might have been from two different harvests with different climate conditions. This is important since the climate has an impact on the phenolic content and the antioxidant capacity [54]. Thus, and since polyphenols have many beneficial effects on health, their high contents in the 0% DCJ might explain the fact that there was poor evidence for the induction of intestinal inflammation after consumption of this DCJ. Anthocyanins and PACs have already been associated with protection against intestinal inflammation by improving the intestinal environment and reducing some biomarkers of inflammation [27,47,55,56,57,58,59,60,61,62,63,64,65]. Furthermore, the particularity of PACs from CJ is that there are two existing types of linkages between catechin and epicatechin units: the more common is the B-type linkage (C_4_-C_8_ or C_4_-C_6_) and the less common is the A-type linkage (B-type linkage with a C_2_-O-C_7_ linkage) [13]. Thus, the occurrence of a type over another, might also have played a role in the differences observed between the results of the two studies. Finally, the sum of the total content of anthocyanins and PACs represented 58.5% of the total polyphenol content of the 1:1 CJ and 47.2% of the 0% DCJ. This is interesting since CJ is composed of many other polyphenols, in lower concentrations than anthocyanins and PACs, and they could have a major effect on the protection against potential intestinal inflammation. Thus, it could be hypothesized that there exists a ratio in the content in organic acids/polyphenols contained in CJ modulating intestinal inflammation when daily consumed. The fact that the composition of CJ is harvest-dependent represents a limitation for the study since the composition of CJ changed between studies as well as for the results.

To our knowledge, the impacts of a daily consumption of DCJs on the composition and the functions of the gut microbiota was investigated for the first time. First, the effect of DCJ administration to mice was compared to the control group receiving water. The results of LEfSe analysis demonstrated that the 0, 60, and 80% DCJs induced changes in the gut microbiota composition. The high polyphenol content of these DCJs, which was the same, could probably induce the increased presence of *Lachnospiraceae_NK4A136_g* as already observed with a polyphenol-rich cranberry extract, a bilberry anthocyanin extract, and a flavonoid-rich green seaweed extract [27,66,67]. Bacteria from *Lachnospiraceae* family are known as Short Chain Fatty Acid (SCFA) producers which contribute to the energy source of epithelial cells and exert anti-inflammatory effects, thus allowing to explain our previous results on intestinal inflammation [55,66,68]. Furthermore, the results of relative abundance demonstrated an increased abundance of *Lachnospirales* whose family of *Lachnospiraceae* belongs, corroborating the results of LEfSe. Additional experiments could be done in a future study, to determine SCFA content in the cecum or in the feces of mice in order to validate such results. Furthermore, since the feces were pooled by cage for this experiment thus, decreasing the statistical power, it would be interesting to statistically analyze the relative abundance of taxa between groups in a future study. Additionally, the 0 and 60% DCJs induced an increased presence of *Coriobacteriales_Incertae_Sedis_*g and this could be correlated to the content in polyphenols of the juice since wild blueberry PACs with a degree of polymerization of 4 and extracts, increased its presence in High-Fat/High-Sucrose (HFHS)-fed mice [69]. Furthermore, the *Coriobacteriales* family is known as a degrading polyphenol family and since the availability of polyphenols is limited in the intestine, the increased abundance of bacterium from this family could increase the polyphenol absorption through the epithelial, thus leading to beneficial health effects. Additional experiments could be undertaken to measure the absorption of polyphenols in the gut of mice. Finally, the administration of the different DCJs induced alterations in the functions of the gut microbiota of mice. As the rate of deacidification of CJ increased, the number of functions also increased as much in the control group as the DCJ-treated groups. The increase in abundance of functions could be correlated to the increased abundance of bacteria from *Lachnospiraceae* family as observed in Figure 3C and Figure 4C,D. Indeed, the abundances of pathways calculated by PICRUSt2 are directly correlated to the abundance of the bacteria identified in the composition of the gut microbiota.

**Figure 8:**
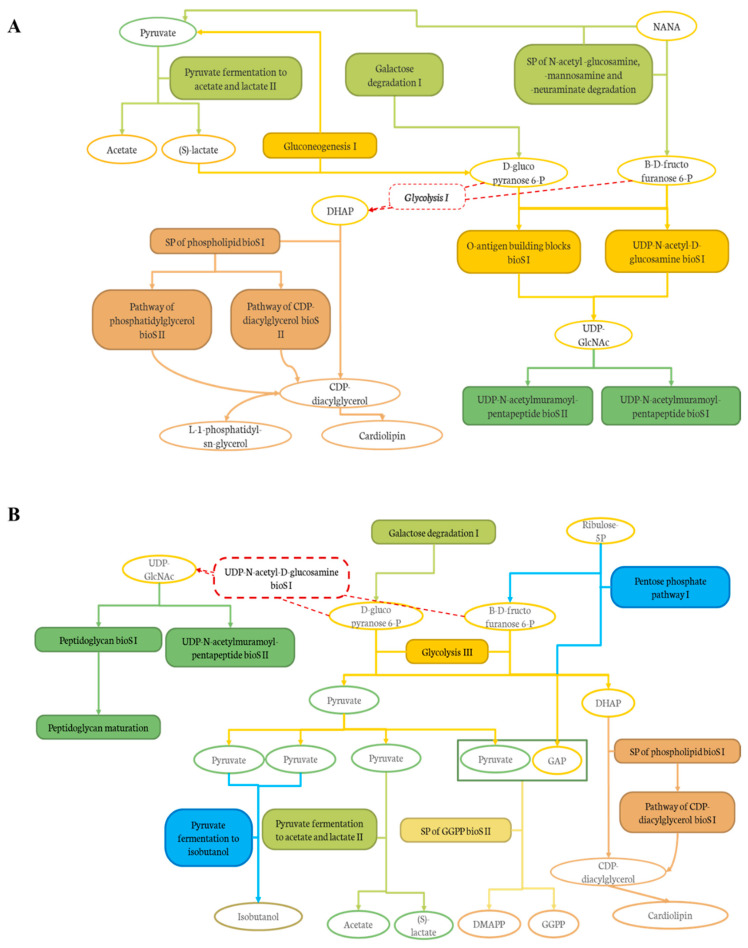
Graphical representation of the links between the pathways and SP induced by (**A**) the 60% DCJ and (**B**) the 80% DCJ in the gut microbiota of the treated mice in comparison with a 0% DCJ. Filled frames represent degradation (light green), sugar biosynthesis (dark yellow), pentapeptide biosynthesis (dark green), lipid biosynthesis (brown), generation of precursor metabolites and energy (blue), and polyprenyl biosynthesis (light yellow).

In addition, the effect of the level of deacidification of CJ was compared to the 0% DCJ-treated group to evaluate the impact of organic acid removal on the composition and functions of the gut microbiota. The results indicated that the 60 and 80% DCJs induced an increased abundance and members of the *Lachnospiraceae* family other than the unidentified genus *Lachnospiraceae_NK4A136_g*. As already explained, this was probably due to the high polyphenol content of these DCJs, but this was interesting since the juices compared here were composed of the same content of polyphenols. Thus, it could be hypothesized that the overrepresentation of these bacteria could be correlated to the content in organic acids of the DCJs. Indeed, the lower the organic acid content, the more bacteria of the *Lachnospiraceae* family there was. Furthermore, there was an overrepresentation of *Lachnoclostridium* in the gut microbiota of the 80% DCJ-treated group and it could be hypothesized that this bacterium is more sensitive to the content in organic acids of the DCJ as already observed with aronia polyphenols [70]. Additionally, there was an impact of the level of deacidification of CJ on the functions of the gut microbiota of mice. The results indicated that the 60 and 80% DCJs induced an increased abundance of specific functions in the gut microbiota of the treated mice compared to the 0% DCJ-treated mice. It was interesting since some of the pathways and superpathways (SP) could be associated as presented in Figure 8A,B, thus, giving a better understanding of the action of the DCJs. As observed in Figure 8A, the 60% DCJ could induce the degradation of N-acetyl-neuraminate (NANA), known to be an energy source for pathogens, into pyruvate, itself fermented into SCFAs, which are energy source for the epithelial cells [71,72]. The further productions of D-glucopyranose 6-phosphate and β-D-fructofuranose 6-phosphate lead to the final activation of pentapeptide biosynthesis (bioS) pathways which in turn, produces peptidoglycans, a major component of cell/bacterium wall. Glycolysis I was not modulated by the 60% DCJ but it is a normal occurring mechanism in the gut and could lead to the further production of dihydroxyacetone phosphate (DHAP), thus, leading to the production of lipids such as phosphatidylglycerols, constituents of the membrane of some bacteria [73]. Regarding Figure 8B, as for the 60% DCJ, the 80% DCJ could also induce the production of SCFAs, lipids, pentapeptides, and peptidoglycans. Furthermore, this DCJ could induce the activation of a pathway for the generation of precursor metabolites and energy such as pyruvate fermentation to isobutanol, and the production of polyprenyl type molecules. GGPP is known as a precursor of different terpenoid class biosynthesis, related to potential health effects [74,75]. Here again, it seems that there was a correlation between the content in organic acids of the DCJ and the number and type of functions exhibited by the gut microbiota. Indeed, the lower the organic acid content, the more representation of functions that seemed to have a positive impact on the gut microbiota and possibly on the host.

## 4. Materials and Methods

### 4.1. Cranberry Juice

#### 4.1.1. Cranberry Juice and Sample Preparation

A 14°B, pasteurized and clarified CJ was provided by Fruit d’Or (Plessiville, Quebec, Canada) and it was diluted with mouse feeding water in order to obtain a 7°B CJ. EDBM was used to remove the organic acid content at different levels and 0, 40, 60, and 80% DCJ were produced. The electrodialysis cell used during this experiment was a pre-industrial EUR2-C cell (Eurodia, Pertuis, France) and the cell configuration and the electrodialytic protocol were similar to those used by Faucher et al. [42]. All DCJs were stored at −20 °C and thawed at 4 °C before each experiment.

#### 4.1.2. Analyses of the Physicochemical Composition of DCJ

##### pH

The pH of DCJ and recovery solutions was measured using a VWR Symphony pH-meter model SP20 (Thermo Orion, West Chester, PA, USA).

##### Conductivity

The conductivity of both DCJ and recovery solutions was measured using an YSI conductivity meter model 3100 (Yellow Springs Instrument, Yellow Springs, OH, USA) connected to an YSI Immersion probe model 3252 (cell constant K = 1.0 cm^−1^). It was expressed in µS/cm.

##### Titratable Acidity

The titratable acidity was evaluated according to AOAC method 942.15. A volume of 4mL of DCJ was diluted with 40 mL of degassed distilled water and 0.1 M NaOH solution was added until pH 8.2 was reached. The titratable acidity was expressed as g/L of citric acid monohydrate equivalent.

##### Organic Acid Contents

The content of quinic, malic, and citric acids in all DCJ was measured by HPLC according to AOAC method 986.13 and following the protocol of Renaud et al. [24]. Each organic acid content was expressed in mg/L.

##### Polyphenol Contents

The anthocyanin and PAC contents in DCJ were measured by HPLC following the protocols of Renaud et al. [24]. Each anthocyanin content was expressed in mg/L of cyanidin equivalents while proanthocyanidin content was expressed in mg/L of epicatechin equivalents.

The total polyphenol content was measured using microscaled Folin–Ciocalteau assay with a xMark Microplate spectrophotometer (Bio-Rad Laboratories Inc., Hercules, CA). The detection wavelength was set at 765 nm and total polyphenol concentration was expressed in mg/L of gallic acid equivalent.

### 4.2. Animals

#### 4.2.1. Animals and Dietary Treatments

Sixty C57BL/6J female mice (6-week-old) were purchased from the Jackson Laboratory (Bar Harbor, ME, USA) and housed in groups (*n* = 3 in each cage) at an ambient temperature of 25 °C, 12 h/12 h of light and dark cycle with *ad libitum* access to food and water. After two weeks of acclimation, mice were divided in 5 groups (control (water), 0, 40, 60, and 80% DCJ) of 12 mice according to their initial weight (between 17.3 and 17.5g per group). All groups were fed a standard normal CHOW diet and received an oral daily dose of 200 µL/25 g of body weight, of water for the control group or one of the 4 different levels of DCJ, for 4 weeks. The volume of water and/or DCJ was adjusted twice every week according to the weight of each mouse in order to keep the maximal dose given to them. Data such as mouse weight and food consumption/cage were collected 2 times a week. Fresh feces were collected in two sterile tubes before the beginning of the experiment (T0), after 2 weeks of gavage (T2), and before their sacrifice (T4). One tube of fresh feces was used to reveal the presence of OB with a Hemoccult test. The other tube was used to analyze the gut microbiota of mice. After the 4 week study, animals were anesthetized in chambers saturated with 2–3% isoflurane and then sacrificed by cardiac puncture. Organs such as liver, spleen, ileum, and colon were collected and kept for further analyses. The protocol was approved by the Laval University Animal Ethics Committee (number 2018072).

#### 4.2.2. Analyses Determining Intestinal Inflammation

##### Occult Blood Testing

The presence of OB in fresh feces samples was revealed using an Hemoccult SENSA kit (Beckman Coulter, CA, USA), per manufacturer’s guide.

##### Intestine Macroscopic Observations

Inspired from the existing literature, a severity score of intestinal inflammation was assessed by macroscopic observations of the ileum and the colon of mice [40,41]. In this purpose, the intestine of mice was removed and starting from the caecum, 3 cm of the ileum and colon were isolated and gently washed in cold 1× Phosphate-Buffer Saline (PBS). Then, a score value was established by observing the extent (in cm) of 3 criteria: inflammation (reddish zone), vascularization, and thickening (Table 3). Finally, the severity score of intestinal inflammation was calculated based on the score values of the 3 criteria and ranged between 0 and 3. Once the observations were done, 1 cm of each colon and ileum was kept in RNA later for measurement of gene expression by qRT-PCR.

##### Gene Expression Analyses by qRT-PCR

The gene expression of *Tnf*, *Muc2*, and *IL22* was analyzed in the colon of mice as followed and per each manufacturer’s guide. First, the E.Z.N.A Total RNA kit I (Omega bio-Tek, Norcross, GA, USA) was used to extract the total RNA of the colon. Then, a Nanodrop spectrophotometer ND-1000 (Thermo Fisher Scientific, Carlsbad, CA, USA) and an Agilent 2100 Bioanalyzer (vB.02.08.SI648), an Agilent RNA 6000 Nano Kit, and RNA NanoChips (Agilent Technologies, Waldbronn, Germany) were used to determine the concentration of total RNA and check its quality. The SuperScript III First-Strand Synthesis SuperMix (Thermo Fisher Scientific, Carlsbad, CA, USA) was used to the transcription of the mRNA of total RNA samples into cDNA. The amplification of the resulting cDNA by qRT-PCR was performed by using of SYBR Green Fast Mix Low Rox (Quanta bio, Beverly, MA, USA) on a Mx 3000P thermocycler (Agilent, Santa Clara, CA, USA). The PCR cycling conditions were 95 °C for 10 min, followed by 40 cycles of 95 °C for 15 s and 60 °C for 1 min. Primer sets, RT2 qPCR were purchased from Qiagen (Hilden, Germany) (Table S2). Finally, the quantification of the expression of the gene of interest were analyzed with a MxPro program (Agilent, Santa Clara, CA, USA), the relative transcripts levels were normalized to the housekeeping controls PeptidylProlyl Isomerase A (*Ppia*) and Glucuronidase β (*Gusb*) and expressed as the percentage of the gene expression.

#### 4.2.3. Bioinformatics Analyses Determining the Modulation of Gut Microbiota

##### Fecal Sample Processing and 16S rRNA Gene-Based Sequencing

Fresh feces collected at T0 and T4 were used for the analysis of the gut microbiota. Bacterial DNA was extracted from the collected samples and purified using a NucleoSpin® 96 Soil kit (Macherey-Nagel, Germany), in accordance with the manufacturer’s guide. The V3-V4 region of the 16S rRNA gene of the DNA extracts was then amplified using 341F (5′-CCTACGGGNGGCWGCAG-3′) and 805R (5′-GACTACHVGGGTATCTAATCC-3′) primers, which are adapted to incorporate the transposon-based Illumina Nextera adapters (Illumina, San Diego, CA, USA) and a sample barcode sequence, both of which allow for multiplexed paired-end sequencing. The amplification mix contained 1× Q5 buffer (NEB), 1× Q5 Enhancer (NEB), 200 μM dNTP (VWR International, Mississauga, Ontario, Canada), 0.2 μM of forward and reverse primers (Integrated DNA Technologies, Coralville, IA, USA), 1 U of Q5 (NEB), and 1 μL of template DNA in a 50 μL reaction. The cycling condition was as follows: denaturation (30s at 98 °C), followed by a first set of 15 cycles (98 °C for 10 s, 55 °C for 30 s, and 72 °C for 30 s), a second set of 15 cycles (98 °C for 10 s, 65 °C for 30 s, and 72 °C for 30 s), and a final elongation period (2 min at 72 °C). The construction and sequencing of 16S rRNA gene-based libraries were performed as previously described [76].

##### Gut Microbiota Analyses

Forward and reverse primers were removed from raw paired-end reads using Cutadapt (v1.14; [77]). Sequences were then demultiplexed, denoised, dereplicated, and merged with the DADA2 package in the R software (vR.3.6.0) [78]. The resulting amplicon sequence variants (ASVs) table was assigned taxonomy using the Ribosomal Database Project RDP classifier algorithm (v2.2; [79]), trained against the SILVA database 138 [80]. Taxa that appeared less than three times in the entire dataset were removed. Samples were rarefied to an even sampling depth of 20,296 sequences per sample.

##### Functional Prediction of Gut Bacterial Communities

Prediction of functional genes was performed using KEGG 3 (Kyoto Encyclopedia of Genes and Genomes) and PICRUSt2 [81]. Briefly, the ASVs table was used to predict the MetaCyc pathway abundances using picrust2_pipeline.py as provided (https://github.com/picrust/picrust2/wiki/Full-pipeline-script) [82].

### 4.3. Statistical Analyses

The data were subjected to an analysis of variance (ANOVA), Tukey tests were carried out to evaluate the composition of the DCJ, the physiological parameters, and the post-mortem observations. A t-test was performed to compare the gene expression of control and treated groups. For this statistical analytic purpose, SAS software (v6.1) was used and significant differences were declared at a probability level *p* < 0.05.

Regarding gut microbiota data, the detection of differentially abundant taxa or pathways between groups was performed with the metagenomics biomarker discovery tool for LeFSe using an LDA score threshold ≥2.5 or ≥2.0, respectively [83]. Differences in beta-diversity were visualized with principal coordinate analysis (PCoA) based on the unweighted UniFrac (Unique Fraction) distance, as described previously [84].

## 5. Conclusions

This study demonstrates for the first time the beneficial effects of the deacidification of CJ by EDBM on the gut microbiota. The modulation of the composition and the functions of the gut microbiota according to the level of deacidification of this juice led to innovative knowledge in the field of health as well as the applicability of the EDBM process in the juice industry. The market for such a new deacidified CJ is estimated at more than USD 1.0 million per year. Furthermore, this study could improve the industrial methods. Indeed, by measuring the organic acid content and polyphenol content of CJ, the most irritant CJs for consumers could be identified and selected for further deacidification by EDBM.

Additionally, for future studies, it could be interesting to investigate the effect of such DCJs (0, 40, 60, and 80%) in induced intestinal inflammation model mice since most of the studies found in the literature were carried out on this type of model and highlighted the protective effect of the polyphenols on intestinal inflammation. Furthermore, it could also be interesting to determine precisely the levels of organic acids, anthocyanins and PACs contained in a CJ, initiating intestinal inflammation. Finally, it could be relevant to study the effect of such DCJ in a clinical trial, since it has never been carried out.

## Figures and Tables

**Table 1:** Physicochemical characteristics of the 0, 40, 60, and 80% DCJs. Data on the same line with different letters are significantly different at a probability level of 0.05.

CJs	0% DCJ	40% DCJ	60% DCJ	80% DCJ
**Deacidification (%)**	0	42	60	79
pH	2.59 ± 0.03 ^a^	2.71 ± 0.01 ^b^	2.87 ± 0.01 ^c^	3.24 ± 0.02 ^d^
Conductivity (µS/cm)	2 409.00 ± 5.00 ^a^	1 883.00 ± 15.72 ^b^	1 966.67 ± 15.89 ^d^	1 840.00 ± 4.36 ^c^
Titratable acidity (g of citric acid eq/L)	9.25 ± 0.05 ^a^	5.40 ± 0.05 ^b^	3.72 ± 0.02 ^c^	1.91 ± 0.05 ^d^
°Brix	6.90 ± 0.00 ^a^	6.10 ± 0.00 ^b^	5.80 ± 0.00 ^c^	5.60 ± 0.00 ^d^
**Organic acids (g/L)**				
Quinic	10.35 ± 0.31 ^a^	10.49 ± 0.17 ^a^	10.11 ± 0.14 ^a^	9.19 ± 0.11 ^b^
Malic	6.03 ± 0.10 ^a^	2.40 ± 0.11 ^b^	1.34 ± 0.06 ^c^	0.00 ± 0.00 ^d^
Citric	11.59 ± 0.20 ^a^	6.88 ± 0.15 ^b^	4.67 ± 0.09 ^c^	2.35 ± 0.06 ^d^
**Anthocyanins (mg of cyanidin eq/L)**				
C-3-galactoside	65.14 ± 0.51 ^a^	64.70 ± 0.32 ^a^	65.76 ± 1.22 ^a^	61.89 ± 0.61 ^b^
C-3-glucoside	2.15 ± 0.12 ^ab^	2.47 ± 0.10 ^b^	2.06 ± 0.06 ^a^	2.22 ± 0.19 ^ab^
C-3-arabinoside	51.12 ± 0.69 ^a^	50.99 ± 0.37 ^a^	50.57 ± 0.89 ^a^	48.24 ± 0.22 ^b^
P-3-galactoside	84.74 ± 0.54 ^a^	83.76 ± 0.51 ^a^	85.02 ± 1.21 ^a^	80.74 ± 0.71 ^b^
P-3-glucoside	8.50 ± 0.10 ^a^	9.01 ± 0.12 ^b^	8.71 ± 0.06 ^ab^	8.53 ± 0.10 ^a^
P-3-arabinoside	37.94 ± 0.62 ^a^	37.13 ± 0.36 ^a^	37.29 ± 0.34 ^a^	35.98 ± 0.30 ^b^
Total	249.58 ± 2.42 ^a^	248.05 ± 0.67 ^a^	249.40 ± 3.88 ^a^	237.60 ± 1.97 ^b^
**PACs (mg of epicatechin eq/L)**				
Monomers	39.35 ± 0.64 ^a^	36.05 ± 2.74 ^a^	36.67 ± 2.41 ^a^	37.43 ± 1.97 ^a^
2–3 mers	148.36 ± 1.80 ^a^	142.12 ± 18.47 ^a^	157.39 ± 3.86 ^a^	159.30 ± 9.67 ^a^
4–6 mers	59.92 ± 1.24 ^a^	57.64 ± 7.06 ^a^	62.55 ± 1.88 ^a^	62.29 ± 3.58 ^a^
7–10 mers	4.28 ± 0.27 ^a^	4.06 ± 0.54 ^a^	4.41 ± 0.48 ^a^	4.53 ± 0.50 ^a^
Polymers	5.55 ± 0.52 ^a^	5.90 ± 0.38 ^a^	5.88 ± 0.05 ^a^	5.78 ± 0.05 ^a^
Total	257.46 ± 2.36 ^a^	245.78 ± 29.00 ^a^	266.91 ± 8.35 ^a^	269.33 ± 15.61 ^a^
**Total polyphenols (mg of gallic acid eq/L)**	1 074.79 ± 4.90 ^a^	978.42 ± 47.79 ^b^	1 075.52 ± 34.87 ^a^	984.21 ± 53.66 ^b^

**Table 2:** Effect of the deacidification of CJ on the inflammatory estate of (**A**) the ileum and (**B**) the colon. Results are presented as means ± SD for each group at each column (*n* = 12). The last column indicates the final score for each group and a different letter in this column indicates a statistically significant difference between the groups at a probability level of *p* < 0.05.

**(A)**				
**Groups**	**Inflammation**	**Vascularization**	**Thickening**	**Total Score**
Control	0.00 ± 0.00 ^a^	0.08 ± 0.29 ^a^	0.00 ± 0.00 ^a^	0.03 ± 0.17 ^a^
0% DCJ	0.25 ± 0.87 ^ab^	1.42 ± 1.5 ^b^	0.00 ± 0.00 ^a^	0.56 ± 1.16 ^ab^
40% DCJ	1.27 ± 1.49 ^bc^	0.73 ± 0.90 ^a^	0.00 ± 0.00 ^a^	0.67 ± 1.11 ^b^
60% DCJ	1.42 ± 1.51 ^c^	0.58 ± 1.16 ^a^	0.00 ± 0.00 ^a^	0.67 ± 1.22 ^b^
80% DCJ	0.00 ± 0.00 ^a^	0.17 ± 0.58 ^a^	0.00 ± 0.00 ^a^	0.06 ± 0.33 ^a^
**(B)**				
**Groups**	**Inflammation**	**Vascularization**	**Thickening**	**Total Score**
Control	0.00 ± 0.00 ^a^	0.08 ± 0.29 ^a^	0.00 ± 0.00 ^a^	0.08 ± 0.29 ^a^
0% DCJ	0.25 ± 0.45 ^a^	0.08 ± 0.29 ^a^	0.33 ± 0.65 ^a^	0.67 ± 0.89 ^a^
40% DCJ	0.45 ± 0.93 ^a^	0.09 ± 0.30 ^a^	0.27 ± 0.65 ^a^	0.82 ± 1.40 ^a^
60% DCJ	0.67 ± 0.89 ^a^	0.50 ± 0.67 ^b^	0.25 ± 0.45 ^a^	1.42 ± 1.38 ^a^
80% DCJ	0.25 ± 0.45 ^a^	0.08 ± 0.29 ^a^	0.25 ± 0.45 ^a^	0.58 ± 0.90 ^a^

**Table 3:** Macroscopic observations scoring of the ileum and colon of mice.

Score	Inflammation	Vascularization	Thickening
0	None	None	None
1	0.1 to 1 cm	0.1 to 1 cm	0.1 to 1 cm
2	1.1 to 2 cm	1.1 to 2 cm	1.1 to 2 cm
3	2.1 to 3 cm	2.1 to 3 cm	2.1 to 3 cm

## Data Availability

Data is contained within the article.

## Supplementary Materials

The following are available online at https://www.mdpi.com/article/10.3390/ijms222111537/s1.
