# Supplementary material for: The Concentration of Organic Acids in Cranberry Juice Modulates the Gut Microbiota in Mice
Source: Int J Mol Sci. 2021 Oct 26;22(21):11537. doi: 10.3390/ijms222111537 (PMC8584276; doi:10.3390/ijms222111537)
Supplement: Supplementary file 1 [file ijms-22-11537-s001.zip › ijms-1396579-supplementary.pdf]

Supplementary material

Table S1. Comparison of the physicochemical characteristics of the 1:1 CJ [25] and the 0% DCJ of the current study. The column “% variation” corresponds to the variation of values of the 0% DCJ compared to the 1:1 CJ. A different letter on the same line mean that there is a statistically significant difference between the juices at a probability level of 0.05.

**Cranberry juices**	**1:1 CJ**	**0% DCJ**	**% variation**	**p-value**
**pH**	2.51 ± 0.02[a]	2.59 ± 0.03[b]	↗ 3.1	0.0154
**Titratable acidity (g of citric acid eq/L)**	9.49 ± 0.06[a]	9.25 ± 0.05[b]	↘ 2.5	<0.0001
**°Brix**	7.37 ± 0.06[a]	6.90 ± 0.0[b]	↘ 6.4	0.0002
**Organic acids (mg/L)**				
Quinic	10 710.76 ± 31.53[a]	10 354.84 ± 306.22[a]	↘ 3.3	0.1157
Malic	6 571.50 ± 72[a]	6 029.82 ± 101.43[b]	↘ 8.2	0.0017
Citric	12 467.90 ± 511.09[a]	11 589.89 ± 204.20[a]	↘ 7.0	0.0501
**Anthocyanins (mg of cyanidin eq/L)**				
C-3-galactoside	17.10 ± 0.35[a]	65.14 ± 0.51[b]	↗ **73.8**	<0.0001
C-3-glucoside	2.80 ± 0.19[a]	2.15 ± 0.12[b]	↘ 23.2	0.0074
C-3-arabinoside	33.81 ± 1.12[a]	51.12 ± 0.69[b]	↗ 33.9	<0.0001
P-3-galactoside	25.02 ± 0.81[a]	84.74 ± 0.54[b]	↗ **70.5**	<0.0001
P-3-glucoside	7.62 ± 0.41[a]	8.50 ± 0.10[b]	↗ 10.4	0.0246
P-3-arabinoside	23.31 ± 0.17[a]	37.94 ± 0.62[b]	↗ **38.6**	<0.0001
Total	109.66 ± 2.07[a]	249.58 ± 2.42[b]	↗ **56.1**	<0.0001
**Proanthocyanidins (mg of epicatechin eq/L)**				
Monomers	49.41 ± 0.68[a]	39.35 ± 0.64[b]	↘ 20.4	<0.0001
2-3mers	139.82 ± 0.76[a]	148.36 ± 1.80[b]	↗ 5.8	0.0016
4-6mers	21.4 ± 0.25[a]	59.92 ± 1.24[b]	↗ **64.3**	<0.0001
7-10mers	2.77 ± 0.37[a]	4.28 ± 0.27[b]	↗ 35.3	0.0047
Polymers	18.37 ± 1.38[a]	5.55 ± 0.52[b]	↘ **69.8**	<0.0001
Total	231.77 ± 3.45[a]	257.46 ± 2.36[b]	↗ 10.0	<0.0001
**Total polyphenols (mg of gallic acid eq/L)**	583.48 ± 87.93[a]	1 074.79 ± 4.90[b]	↗ **45.7**	<0.0001

Table S2. Catalog numbers of primer sets used for qRT-PCR (Qiagen).

**Primers**	**Catalog number**
Mouse *Tnf*	PPM03113G
Mouse *Muc2*	PPM24739G
Mouse *IL22*	PPM05481A
Mouse *Ppia*	PPM03717B
Mouse *Gusb*	PPM05490C
